# EMT-related gene classifications predict the prognosis, immune infiltration, and therapeutic response of osteosarcoma

**DOI:** 10.3389/fphar.2024.1419040

**Published:** 2024-08-07

**Authors:** Meng-Pan Li, Si-Ping Long, Wen-Cai Liu, Kun Long, Xing-Hua Gao

**Affiliations:** ^1^ Department of Orthopedics, Guangzhou First People’s Hospital, South China University of Technology, Guangzhou, China; ^2^ Department of Orthopedics, Shanghai General Hospital, Shanghai Jiao Tong University School of Medicine, Shanghai, China; ^3^ The First Clinical Medical College of Nanchang University, Nanchang, China; ^4^ The Fourth Clinical Medical College of Nanchang University, Nanchang, China; ^5^ Department of Orthopedics, Shanghai Sixth People’s Hospital Affiliated to Shanghai Jiao Tong University School of Medicine, Shanghai, China

**Keywords:** osteosarcoma, EMT, prognostic signature, immune infiltration, therapeutic response

## Abstract

**Background:**

Osteosarcoma (OS), a bone tumor with high ability of invasion and metastasis, has seriously affected the health of children and adolescents. Many studies have suggested a connection between OS and the epithelial-mesenchymal transition (EMT). We aimed to integrate EMT-Related genes (EMT-RGs) to predict the prognosis, immune infiltration, and therapeutic response of patients with OS.

**Methods:**

We used consensus clustering to identify potential EMT-Related OS molecular subtypes. Somatic mutation, tumor immune microenvironment, and functional enrichment analyses were performed for each subtype. We next constructed an EMT-Related risk signature and evaluated it by Kaplan-Meier (K-M) analysis survival and receiver operating characteristic (ROC) curves. Moreover, we constructed a nomogram to more accurately predict OS patients’ clinical outcomes. Response effects of immunotherapy in OS patients was analyzed by Tumor Immune Dysfunction and Exclusion (TIDE) analysis, while sensitivity for chemotherapeutic agents was analyzed using oncoPredict. Finally, the expression patterns of hub genes were investigated by single-cell RNA sequencing (scRNA-seq) data analysis.

**Results:**

A total of 53 EMT-RDGs related to prognosis were identified, separating OS samples into two separate subgroups. The EMT-high subgroup showed favourable overall survival and more active immune response. Significant correlations were found between EMT-Related DEGs and functions as well as pathways linked to the development of OS. Additionally, a risk signature was established and OS patients were divided into two categories based on the risk scores. The signature presented a good predictive performance and could be recognized as an independent predictive factor for OS. Furthermore, patients with higher risk scores exhibited better sensitivity for five drugs, while no significant difference existed in immunotherapy response between the two risk subgroups. scRNA-seq data analysis displayed different expression patterns of the hub genes.

**Conclusion:**

We developed a novel EMT-Related risk signature that can be considered as an independent predictor for OS, which may help improve clinical outcome prediction and guide personalized treatments for patients with OS.

## Introduction

Osteosarcoma (OS), growing from osteogenic mesenchymal stem cells, has long been thought to be the most dangerous tumor in teenagers (Brown et al., 2017; Yang et al., 2021). Patients suffering from localized OS have a 5-year survival rate of approximately 65%, in contrast to roughly 20% for those with recurrent and metastatic OS (Miwa et al., 2019). At present, many treatments have been applied for the therapy of patients with OS, including surgery, chemotherapy, and neoadjuvant chemotherapy, but the overall effects are still unsatisfactory due to the emergence of drug resistance and tumor progression (Kager et al., 2017; Zhou et al., 2023). Therefore, elucidating the potential molecular mechanisms in the development of this tumor and finding new therapeutic approaches are especially important for individuals with OS to get more favourable clinical outcomes.

Many studies have reported that epithelial-mesenchymal transition (EMT) is linked to embryonic growth, cancer invasion and metastasis, and drug resistance emergence (Zhang et al., 2021). During this process, epithelial cells develop into mesenchymal cells with the ability to migrate and invade other areas of the body by losing their apical-basal polarity and intercellular adhesion (Cai et al., 2024). Moreover, EMT is abnormally activated, making cancer cells have the invasive phenotype that extend from the original tumor into the circulatory system. This results in increased cell stemness and the ability of tumor cells to resist different types of therapeutics (Derynck and Weinberg, 2019; Bakir et al., 2020; Zhang et al., 2023).

As a new research approach, bioinformatics analysis could be used to further investigate the connection between diseases and cancer-associated gene sets based on polyphyletic data sources (Gong et al., 2018). In recent years, with the rapid development of genomics, a large amount of genetic data has been provided for the diagnosis and prediction of diseases (Sommer et al., 2022). Meanwhile, various bioinformatics tools and public databases have been established successively, and the cross-fusion of different disciplines also makes the research of bioinformatics analysis more in-depth in medicine. These enable researchers to make great progress in the screening and identification of tumor markers, precise molecular typing of tumors and novel targeted therapies (Wang Q. et al., 2020; Lu et al., 2021; Matsuoka and Yashiro, 2024).

Recent research has demonstrated that EMT is connected to the progression of a variety of malignant cancers, including OS (Yang et al., 2013; Shi et al., 2019). For example, a study by Shao et al. (2022) reported that activation of EMT induced by Tetraspanin 7 overexpression could promote the proliferation and metastasis of OS cells. Moreover, Ruh et al. (2021) found that the process of osteoblastic differentiation in OS cells could be blocked by EMT-transcription factor, ZEB1. These results suggest that the EMT signature might be an OS prognostic factor. In this study, we investigated the connection among EMT, immune response and prognosis in OS patients combining with clinical and gene expression information from openly available databases. We also constructed a risk signature to better predict the prognosis of OS patients. Evaluation of therapeutic response in patients with OS was then been carried out, which may provide implications for developing new treatments and making better clinical strategies.

## Materials and methods

### Data acquisition

**FIGURE 1:**
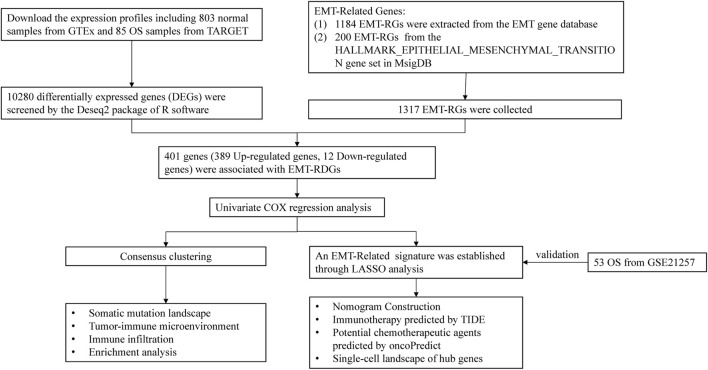
Flowchart of this study.

Figure 1 displays the flowchart for this investigation. Information of OS samples, including RNA sequencing data and clinical characteristics, were extracted from the Therapeutically Applicable Research to Generate Effective Treatments (TARGET; https://ocg.cancer.gov/programs/target) database. Excluding samples that lacked comprehensive clinical information, 85 OS samples were included for further analysis. Gene expression in 803 normal samples was attained from the Genome Tissue Expression (GTEx; https://gtexportal.org/home/datasets) database. Additional 53 OS samples were retrieved from the GSE21257 (https://www.ncbi.nlm.nih.gov/) as the validation cohort.

The EMT-RGs were acquired from the two datasets listed below: 1184 EMT-RGs were download from the EMT gene database (https://www.dbemt.bioinfo-minzhao.org/), and 200 EMT-RGs from the HALLMARK_EPITHELIAL_MESENCHYMAL_TRANSITION gene set in the Molecular Signatures Database (MsigDB; https://www.gsea-msigdb.org/gsea/msigdb). Given that all data in this study was freely accessible online and patients were not involved in the research directly, informed permission and ethical committee approval were not essential.

### Screening EMT-Related DEGs associated with prognosis

The “DESeq2” R package was utilized to compare the gene expression levels of the OS and normal samples, thus screening differentially expressed genes (DEGs). And the thresholds for significance were set to adjust |log2 (FC)| > 2.5 and adjusted *P*-value < 0.01. Then the selected genes were intersected with EMT-RGs to determine the EMT-Related DEGs. By univariate Cox regression analysis, EMT-Related DEGs related to prognosis were screen out for further investigation.

### Consensus clustering and survival analysis

After the identification of prognostic associated EMT-Related DEGs, we identified potential molecular subtypes of the OS in terms of these genes utilizing the ConcensusClusterPlus package in R software. To identify the ideal number of clusters, the k-means clustering method was used for eight cluster numbers k, ranging from 2 to 9, and the procedure was replicated one thousand times to ensure stable outcomes. Then, the K-M survival analysis was employed in order to confirm whether the EMT-Related subtypes had a notable influence on OS patients’ prognosis.

### Somatic mutation landscape

In order to elucidate the notable predictive variances among subtypes from somatic mutation, we acquired data on somatic mutations of OS patients from the Cancer Genome Atlas database (https://portal.gdc.cancer.gov/). Later on, we employed the maftools R package to create waterfall plots to visualize and summarize the mutation landscape of the EMT-Related subgroups.

### Tumor immune microenvironment landscape

In addition, we also attempted to explain the prognostic differences between subtypes from tumor immune microenvironment landscape. The Estimation of Stromal and Immune cells in Malignant Tumor tissues using Expression (ESTIMATE) is often used to assess the existence of stromal cells and immune cells as well as the purity of malignancies in tumor tissues (Yoshihara et al., 2013; Xie et al., 2020). Utilizing the ESTIMATE algorithm, we determined the stromal-, immune-, estimate-scores, and tumour purity of each OS sample. We analyzed the immune checkpoint (ICP) expression levels in order to assess the correlation between EMT-Related genotyping and immunological function. Furthermore, utilizing the CIBERSORT (deconvolution algorithm), 22 different kinds of human immune cells in OS were estimated, and the wilcoxon test was carried out to assess the difference of immune cell composition between EMT-Related genotyping.

### Enrichment analysis landscape

To explain the prognostic differences between subtypes from the landscape of the pathway and functional landscape, we performed functional enrichment analysis in this study. Firstly, we utilized “DESeq2” R package to identify DEGs between the EMT-Related subtypes [log2 (FC) > 2.5, adjusted *P*-value < 0.01]. Then, the screened genes were employed for Gene Ontology (GO) and Kyoto Encyclopedia of Genes and Genomes (KEGG) enrichment analysis. There are three main categories contained in the GO database, including biological process (BP), cellular component (CC), and molecular function (MF). We showed the top five significant terms in BP, CC and MF. KEGG analysis showed all enriched pathways with significant differences. Furthermore, we presented the top five enriched pathways in each subtype by performing Gene Set Enrichment Analysis (GSEA) to determine which pathways were most substantially enriched in each subtype.

### Construction and assessment of an EMT-Related risk signature

Before signature construction, we performed log2 (TPM + 1) on the expression data, and then used the combat function of the limma package to process the debatch effect on the training set TARGET data and the validation set GSE21257 data. In order to avoid the model overfitting, we used a combination of univariate Cox regression and LASSO Cox regression to identify suitable genes for constructing the risk signature. Each sample’s risk score value was determined by the following formula:

$$Risk\;score=\sum_{i=1}^{n}{coef}_{i}\times x_{i}$$

where *coef*_*i*_ denotes the LASSO Cox regression coefficient of the prognosis-related genes, *x*_*i*_ denotes EMT-Related gene expression level, and *n* indicates gene counts. Then, regarding the median risk score, patients in the training (TARGET-OS) and validation cohorts were separated into two risk subgroups (the high- and the low-risk subgroups). The differences of overall survival between the two subgroups were assessed using K-M survival analysis, with the significance of *P-*value < 0.05. Furthermore, we generated ROC curves and calculated the area under the curve (AUC) to assess the overall survival rate at 1-, 3-, and 5-years, thereby evaluating the predictive precision of the risk model.

### Independence evaluation of the risk signature and nomogram construction

In order to ascertain whether the risk signature was independent of other clinical factors, we evaluated the risk model for OS patients using multivariate Cox regression analysis. Moreover, based on risk scores and clinical features, we constructed a nomogram to more precisely quantify the prognosis of OS patients. A score was assigned to a variable (including gender, age, tumor-site, metastatic situation and risk score) in the scoring system of the nomogram, and all the scores from each sample were added together to get the final score. Then, by the function of converting the score to its probability of the result, we could predict the probability of overall survival with each patient (Park, 2018; Liu et al., 2023). A calibration curve was generated to compare the actual and predicted 1-, 3-, and 5-year survival rates of OS patients in the training cohort in order to assess the nomogram model’s predictive performance (45° dotted line represents the greatest prediction).

### Immunotherapy responsiveness and potential chemotherapeutic agents analysis

We performed immunotherapy responsiveness analysis and explored chemotherapeutic agents to further explore the potential treatment measures of OS patients. In the perspective of immunotherapy, we imported the gene expression matrix into the TIDE online database (http://tide.dfci.harvard.edu/) to predict the immune checkpoint blockade (ICB) responses in OS patients, where a lower TIDE score indicated a more favorable immunotherapy response. Moreover, to identify the immune cells that had a significant association with the risk score, spearman correlation analysis was performed to examine the relationship between the risk score and the 22 immune cell scores that the CIBERSORT algorithm estimated.

OncoPredict, an R package designed by Maeser et al. (2021), is often used to predict the sensitivity of patients with cancers to drug therapies. Genomics of Drug Sensitivity in Cancer (GDSC) database (http://www.cancerrxgene.org/downloads), encompassed information of drug sensitivity (IC50) from 1,000 cell lines, facilitating the study of drug reactions and resistance in OS cell lines (Groisberg et al., 2017; Xie et al., 2022). To assess the responsiveness of TARGET-OS samples to drugs, the oncoPredict R package was employed, and the wilcoxon test (*P* < 0.005) was utilized to determine if chemotherapy sensitivity varied between the high and low-risk categories.

### Single-cell RNA sequencing data analysis

Single-cell RNA sequencing data analysis is a ground-breaking technique in cancer research, which allows researchers to study gene expression variations at the single-cell level and determine the composition of tumor cells. Researchers will probably benefit from a thorough analysis of the immune cell composition in OS samples, which will expand their understanding of prognostic biomarkers (Su et al., 2022; Wang et al., 2022; Ji et al., 2023; Liu et al., 2024). For a deeper insight into the OS tumor immune microenvironment, we demonstrated the cellular composition in OS tumor microenvironment through Tumor Immune Single-cell Hub (TISCH; http://tisch.comp-genomics.org/gallery/) online platform. We performed multivariate Cox regression based on the genes screened by LASSO regression to find the hub genes affecting the prognosis of OS. Then, we demonstrated the expression and distribution of these hub genes in each cell of the tumor microenvironment through TISCH platform.

## Results

### Screening EMT-Related DEGs and identifying two EMT-Related subtypes

**FIGURE 2:**
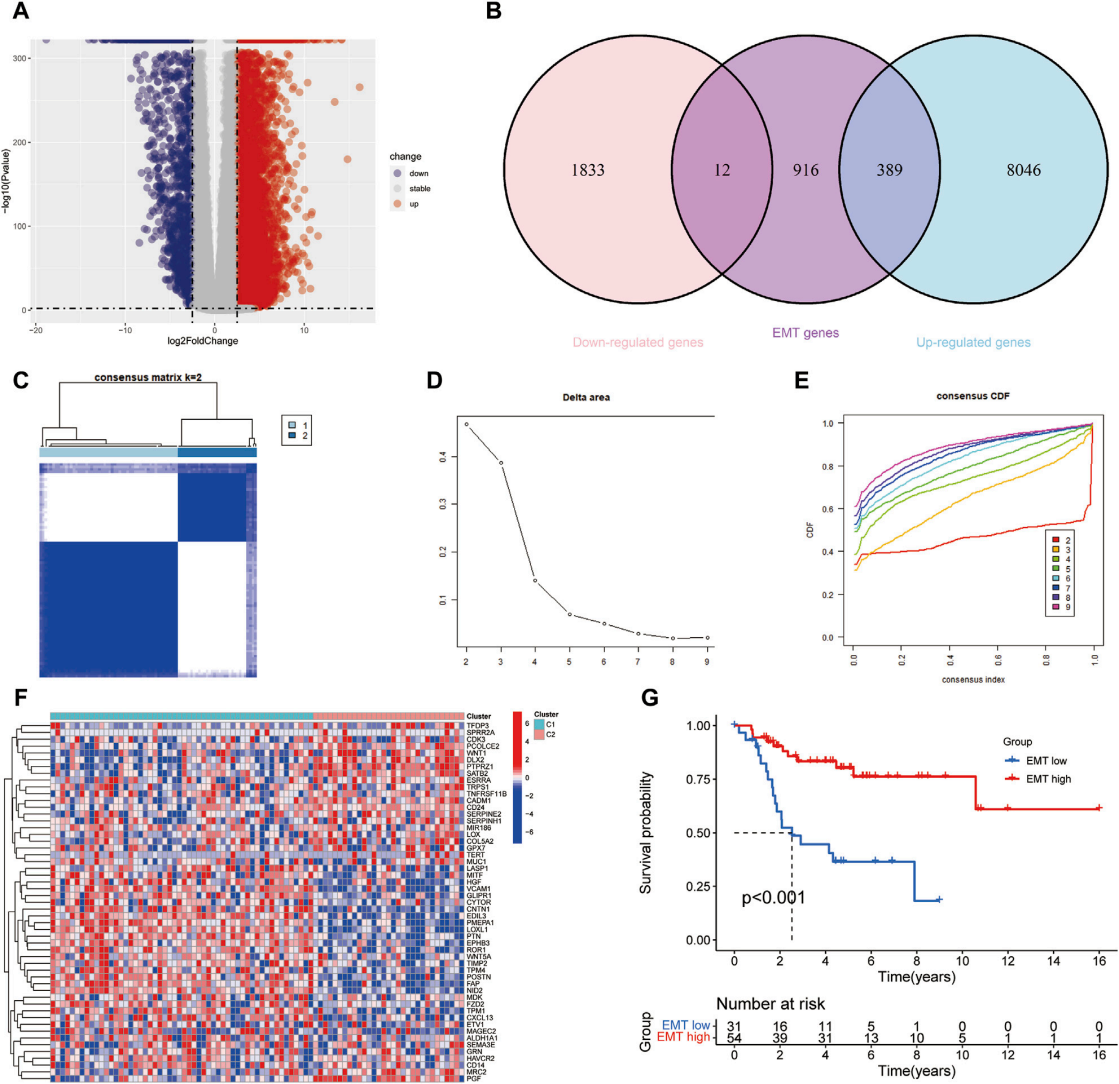
Screening differentially expressed EMT-RGs and identifying potential molecular subgroups. **(A)** Volcano plot showed the Upregulated and Downregulated DEGs between the OS and normal samples. **(B)** Venn diagram displayed the number of EMT-Related DEGs. **(C)** Heatmap showed the consensus clustering solution for 53 EMT-RDGs performed best when k = 2, and OS patients were devided into two clusters. **(D, E)** The consensus clustering delta area curve showed corresponding variations in the area under the cumulative distribution function (CDF) curve for k = 2–9. **(F)** Heatmap displayed the expression of 53 EMT-RDG in the two subtypes. High expression is denoted by red, and low expression is denoted by blue. **(G)** K-M analysis suggested that the EMT-high subgroup manifested a more extended survival period than the EMT-low group, with a notable discrepancy (*P* < 0.001).

We identified 10,280 DEGs between 85 samples and 803 normal samples, of which 8,435 DEGs were Upregulated and 1845 DEGs were Downregulated (Figure 2A). After removing duplicate genes, 1317 EMT-RGs were collected from the two databases. And 401 genes, including 389 Upregulated genes and 12 Downregulated genes, were associated with EMT-RDGs (Figure 2B). Then, using the univariate Cox regression approach, 53 EMT-RDGs related to prognosis were identified for further investigation.

Consensus clustering analysis was carried out to investigate the potential molecular subtypes related to EMT-RDGs in OS, where the number of clusters was denoted by the letter k. When k = 2, the lowest inter-group collinearity and the highest intra-group collinearity was observed. In view of the different EMT-RG expression patterns, the OS samples in the TARGET cohort were divided into two subgroups by k-means clustering (Figures 2C–E). Comparing the gene expression levels of patients in the two cohorts of C1 and C2, they were divided into EMT-high subtype and EMT-low subtype, respectively. Figure 2F demonstrated the expression of 53 EMT-RDGs related to prognosis in the two subtypes. Additionally, K-M survival analysis revealed a noteworthy distinction between the EMT-high and EMT-low groups, with the former showing a longer survival length (*P* < 0.001, Figure 2G).

### Somatic mutation landscape and tumor immune microenvironment in two EMT-Related subtypes

**FIGURE 3:**
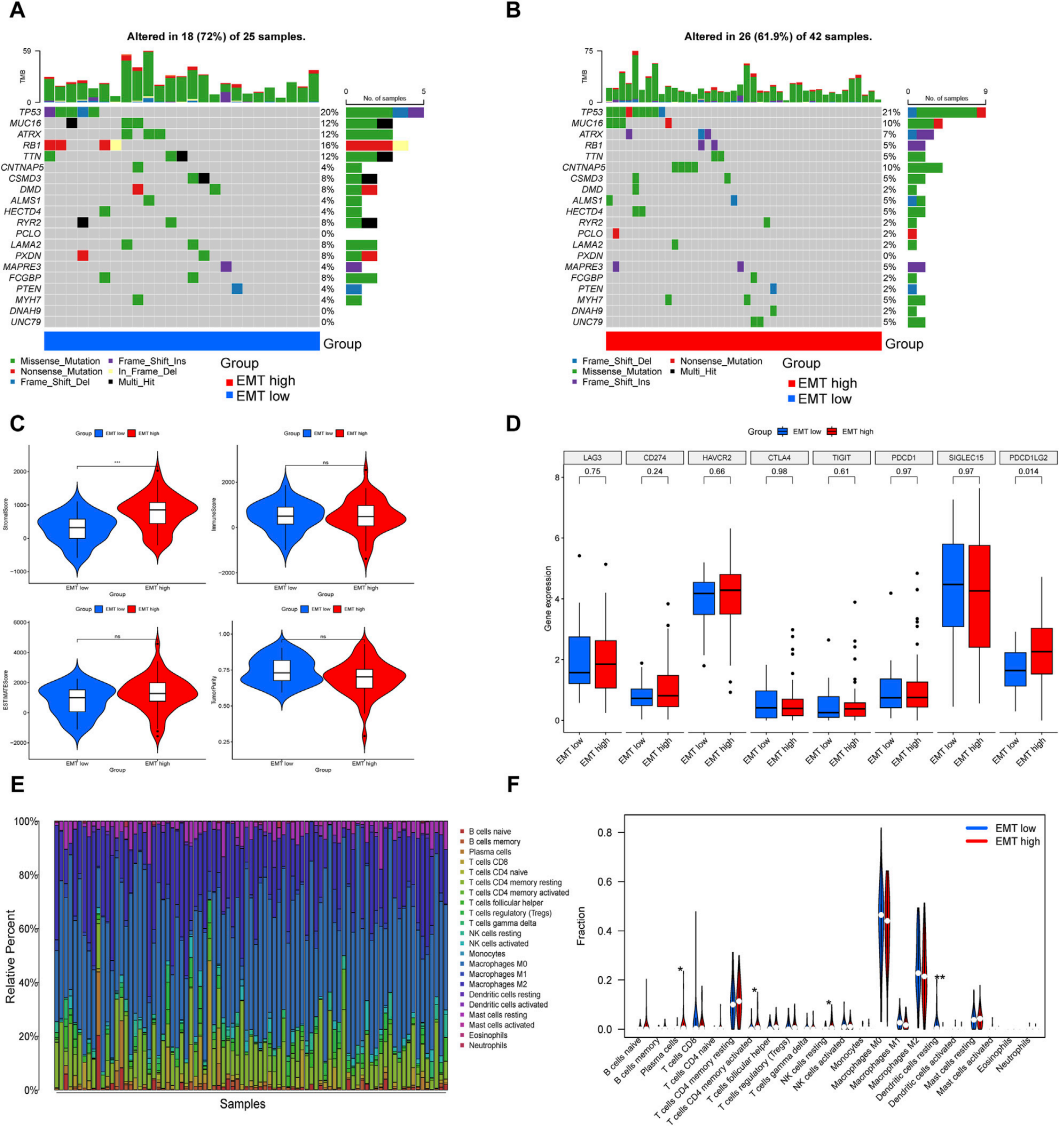
Comparison of somatic mutation landscape and immune landscape in the EMT-high and EMT-low subgroups. **(A, B)** The top 20 genes that were mutated the most often in the two subgroups were displayed in waterfall plots. **(C)** Comparisons between the two subgroups in terms of stromal score, immune score, estimate score, and tumor purity. **(D)** Box plot presented multiple immune checkpoints between the EMT-high and EMT-low subgroups. **(E)** Barplot showed 22 infiltrating immune cells’ composition in each TARGET-OS sample. **(F)** Violin plot illustrated the compositional differences between the two subgroups of the 22 invading immune cells.

In the two gene subtypes, we created waterfall plots in order to visually demonstrate mutated genes (Figures 3A,B). Compared to the EMT-high group, the results presented nine decreased gene mutation frequency in the EMT-low group, including TP53, CNTNAP5, ALMS1, HECTD4, PCLO, MAPRE3, MYH7, DNAH9 and UNC79, and the gene that showed highest frequent mutations in both groupings was TP53.

An increasing body of research indicates that immune cells in the tumor microenvironment play a crucial role in the progression of tumor (Cai et al., 2022). Utilizing the ESTIMATE, we determined the stromal-, immune-, estimate-scores, and tumor purity levels between the two subgroups, finding that the group with higher EMT-Related gene expression had a higher stromal score, while the other three showed no notable distinctions (Figure 3C). ICP expression analysis suggested that PDCD1LG2 was upregulated in the EMT-high subgroup (Figure 3D). Subsequently, we assessed the extent of immune infiltration in 22 different kinds of immune cell types of OS patients in the TARGET database by the CIBERSORT (Figure 3E). In specifics, patients in the EMT-high class showed greater amounts of plasma cells, activated memory CD4 T cells and resting NK cells, whereas the fraction of resting dendritic cells was reduced in the EMT-high subgroup (Figure 3F).

### GO, KEGG and GSEA enrichment analysis

**FIGURE 4:**
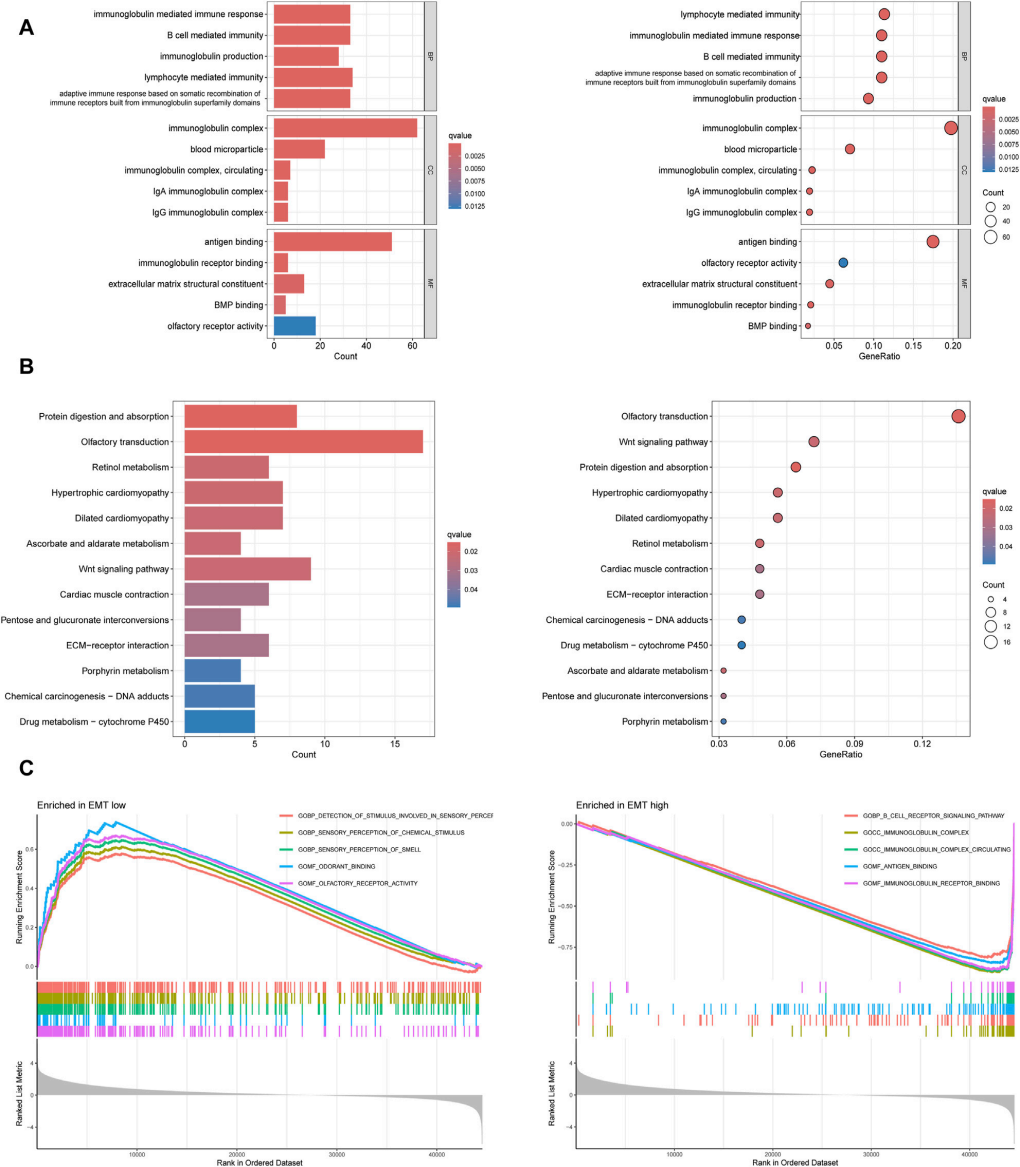
Functional enrichment analyses based on 53 prognosis-related EMT-Related DEGs. **(A)** GO enrichment analysis showed the top five significant terms in BP, CC, and MF. **(B)** KEGG enrichment analysis showed all the pathways with significant differences. **(C)** GO GESA enrichment analysis showed the top five enriched pathways in different subtypes.

GO and KEGG enrichment analysis were conducted based on 53 prognosis-related EMT-Related DEGs to clarify the potential functions and pathways related to EMT-RGs. In GO enrichment analysis, we noted that these genes were positively related with “B cell mediated immunity,” “immunoglobulin mediated immune response,” “immunoglobulin complex,” “antigen binding,” and “immunoglobulin reception binding” (Figure 4A). KEGG analysis revealed three pathways where these genes enriched in, including “wnt signaling pathway,” “potential digestion and absorption,” and “retinol metabolism” and other pathways (Figure 4B). In addition, through GO GSEA enrichment analysis, we found that the main enriched pathways in the EMT-low subgroup included “detection of stimulus involved in sensory perception,” “sensory perception of chemical Stimulus” and “sensory perception of smell.” EMT-high subgroup, on the other hand, were mainly enriched by other pathways including “B cell receptor signaling pathway,” “immunoglobulin complex” and “immnoglobulin complex circulating” (Figure 4C). These enriched terms and pathways might be important in the development of tumor cells.

### Construction of the EMT-Related risk signature

**FIGURE 5:**
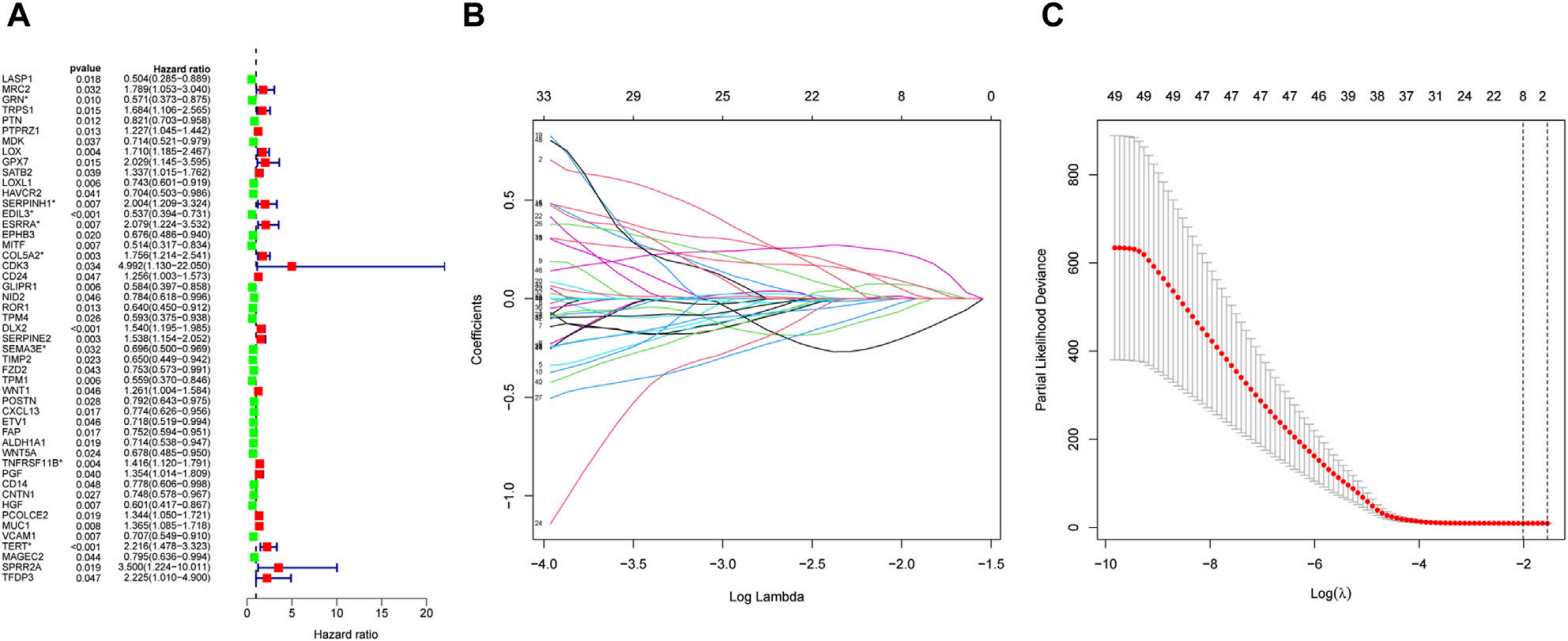
Construction of the EMT-Related risk model. **(A)** 49 EMT-RGs were shown to be connected to OS patients’ overall survival by univariate Cox regression. **(B, C)** Eight prognostic EMT-RGs were screened by LASSO Cox regression and used for constructing the risk signature. “*” was used to highlight the eight genes.

We identified 49 prognosis-related genes that were correlated with OS patient’s overall survival. The expression of 28 genes was linked to extended overall survival of OS patients, whereas 21 genes was linked to reduced overall survival of OS patients (Figure 5A). Eight genes obtained by LASSO analysis as more important genes (including GRN, SERPINH1, EDIL3, ESRRA, COL5A2, SEMA3E, TNFRSF11B, and TERT) were used to establish the risk model (Figures 5B,C).

### Predictive performance evaluation of the EMT-Related risk signature

**FIGURE 6:**
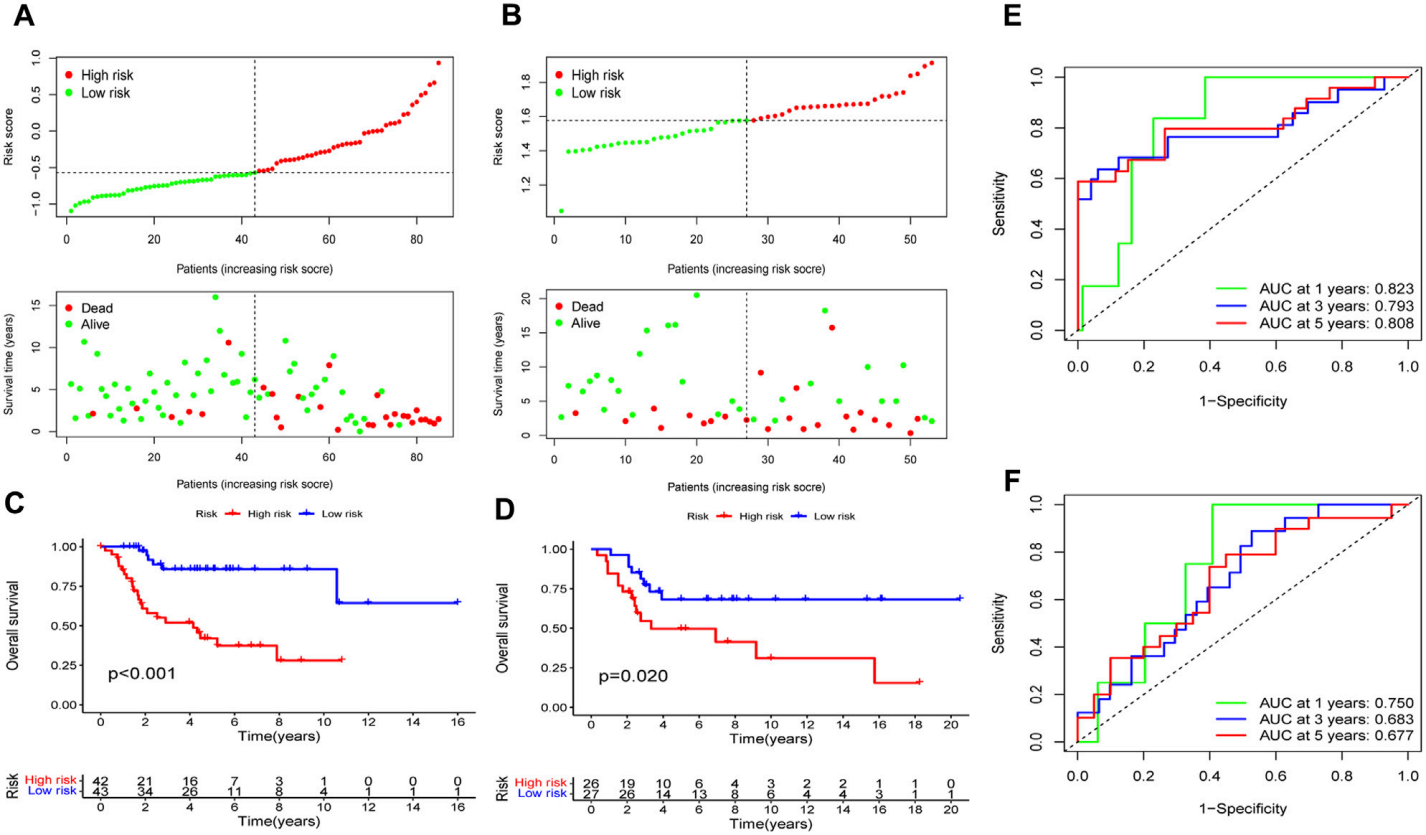
Assess the EMT-Related risk signature’s prediction performance. The training **(A)** and validation **(B)** cohorts of OS patients were classified as low-risk and high-risk subgroups based on the median risk score, and the high-risk group had a higher incidence of deaths in both cohorts. In the training **(C)** and validation **(D)** cohorts, the overall survival rates for OS patients in the high-risk groups was notably lower, according to K-M survival analyses. ROC analyses demonstrated the AUC values of the risk model for 1-, 3-, and 5-year survival rates of OS patients in the training **(E)** and validation **(F)** cohorts.

In the training cohort, the risk model’s prognostic value was initially ascertained, and then verified by the GSE21257 validation cohort. Patients in the training and validation cohorts were separated into the high- and low-risk categories based on the median risk score. The high-risk group had a higher quantity of deaths in both cohorts, indicating poorer prognosis of patients in this group (Figures 6A, B). As shown by the K-M survival analysis, the overall survival rate of the high-risk group of patients was lower than that of the low-risk group (Figures 6C, D). Utilizing ROC analysis, OS patients in the training cohort showed 1-, 3-, and 5-year survival rates with AUC values of 0.823, 0.793, and 0.808, respectively (Figure 6E). Similarly, the AUC values of the risk model were 0.750, 0.683, and 0.677 for the validation cohort at 1-, 3-, and 5-years, respectively (Figure 6F). Collectively, these findings suggest that the risk model demonstrated a high level of predictive accuracy in both the training and validation cohorts.

### EMT-Related risk signature as an independent predictive factor for OS

**FIGURE 7:**
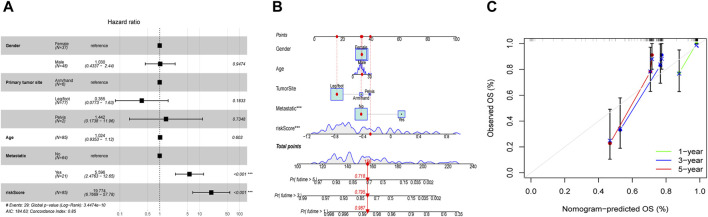
Independent prognostic evaluation and nomogram construction. **(A)** Multivariate Cox analysis demonstrated that the risk score may be used independently to predict OS patients’ prognosis. **(B)** The nomogram for predicting the survival percentage of patients at 1-, 3-, and 5-years in TARGET was constructed using gender, age, tumor-site, metastatic situation, and risk score. **(C)** Calibration curves revealed that there may be a similarity between the nomogram-predicted overall survival of OS patients and their actual survival duration.

Employing the multifaceted Cox regression analysis, the study unequivocally established that patients with OS may be able to use the risk score as an independent predictor of their overall prognosis (Figure 7A). Additionally, to help better predict the clinical outcomes of OS patients, a nomogram was created with the scoring system depicted in the top part and the prediction system in the bottom part (Figure 7B). From Calibration curves, we could find that the predicted survival time could be very close to the actual survival time at 1-, 3-, and 5-years (Figure 7C). The findings above suggested that the EMT-Related risk signature could be considered as an independent predictor of OS and had a lot of promise for therapeutic applications.

### Evaluation of immunotherapy sensitivity

**FIGURE 8:**
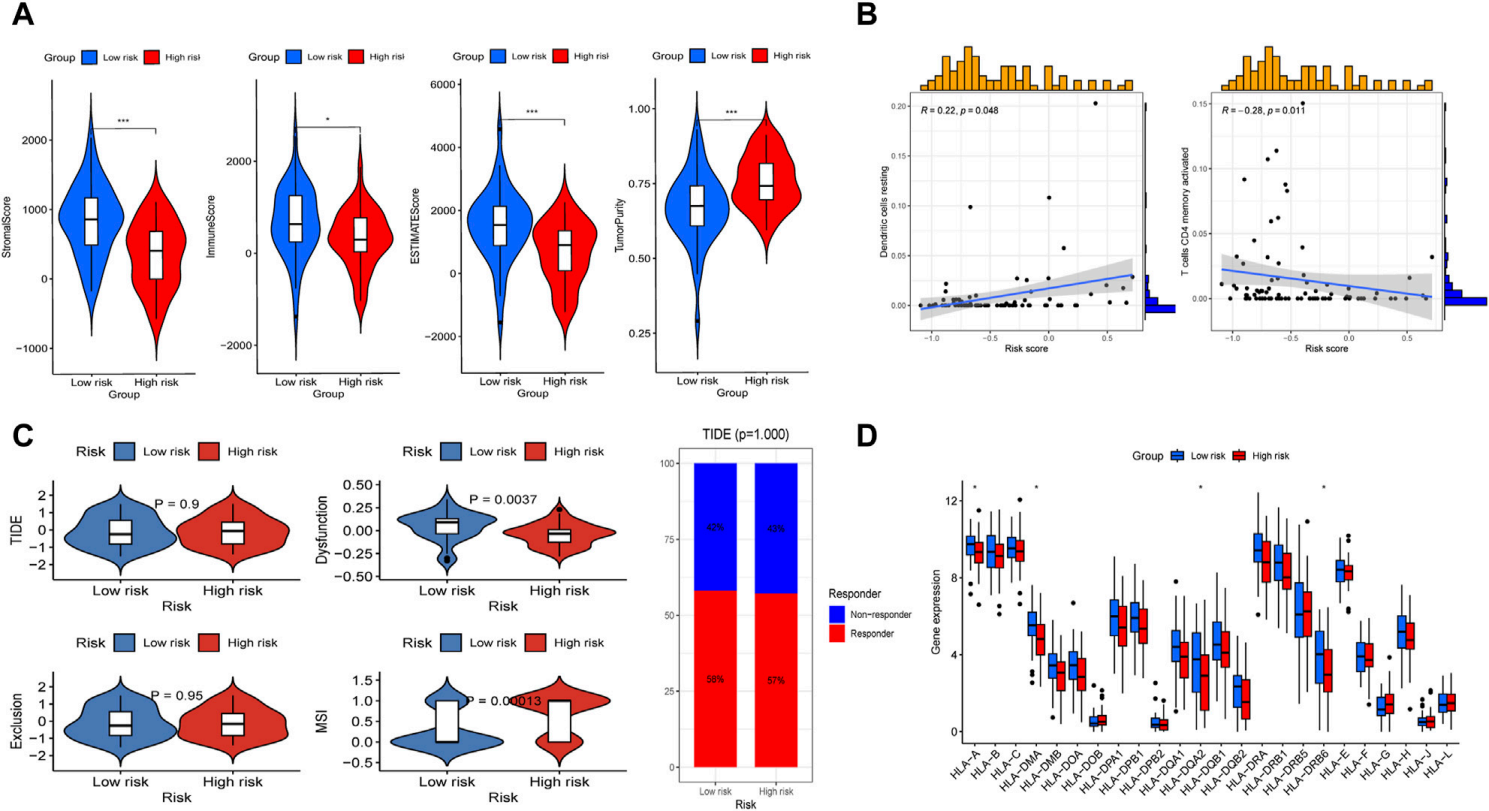
Evaluation of immunotherapy sensitivity in the high-risk and low-risk subgroups. **(A)** Comparisons between the two subgroups in terms of stromal-, immune-, estimate-scores, and tumor purity. **(B)** Spearman correlation study demonstrated the association between immune cells (including resting dendritic cell and active memory CD4 T cells) and risk score. **(C)** The differences of TIDE score, dysfuction score, exclusion score, MSI score, and the proportion of patients whether response to ICP between the two subgroups. **(D)** Box plot presented differential expression of HLA genes between the two subgroups.

We first analyzed the immunological features between the high- and low-risk groups. We found that the low-risk group presented statistically higher stromal score, immune score, estimate score, and lower tumor purity (Figure 8A). Further analysis revealed that there was a positive relationship between the risk and with resting dendritic cell expression (R = 0.22, *P* < 0.05) while a negative relationship existed with activated memory CD4 T cell levels (R = −0.28, *P* < 0.01; Figure 8B). The MSI score was then calculated, and we discovered that the high-risk group had a substantially higher MSI score compared to the low-risk subgroup (Figure 8C). In addition, through TIDE analysis, we found that dysfunction score was decreased in the high-risk group, while TIDE and exclusion scores did not show apparent differences between the two subtypes (Figure 8C). And the percentage of ICB therapy non-responders was similar to that of responders, which indicated that patients with OS may be not sensitive to immunotherapy (Figure 8C). Furthermore, giving the significance of human leukocyte antigen (HLA) genes in anti-cancer immunity, we examined 24 HLA genes across different risk classes. Our findings indicated that most genes were downregulated in the high-risk subgroup (Figure 8D). This result suggested that potential association may existed between the risk score and HLA gene expression levels, which may serve as prospective immunotherapy biomarkers.

### Prediction of potential chemotherapeutic agents

**FIGURE 9:**
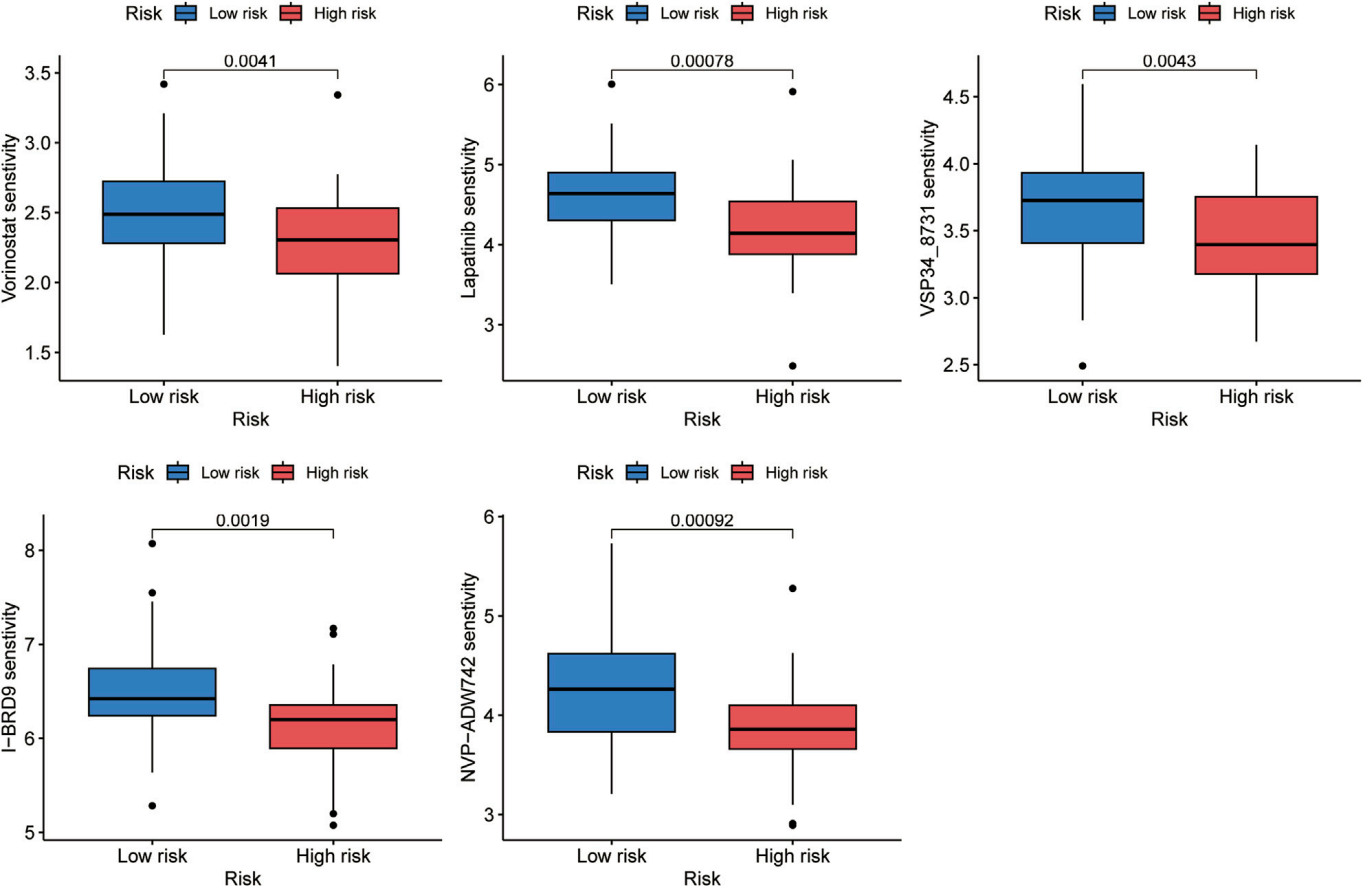
Five chemotherapeutic agents, including vorinostat, lapatinib, VSP34_8731, I-BRD9, and NVP-ADW742, were discovered to be more beneficial for OS patients with higher risk scores.

The correlation between the risk score and sensitivity of some chemotherapeutic agents was calculated by “oncoPredict” package in R software. And the results indicated lower IC50 values and better sensitivity of vorinostat, lapatinib, VSP34_8731, I-BRD9, and NVP-ADW742 in the high-risk group, which implied that aforementioned chemotherapeutic agents would be more beneficial for individuals with higher risk scores (Figure 9).

### Single-cell landscape of hub genes

**FIGURE 10:**
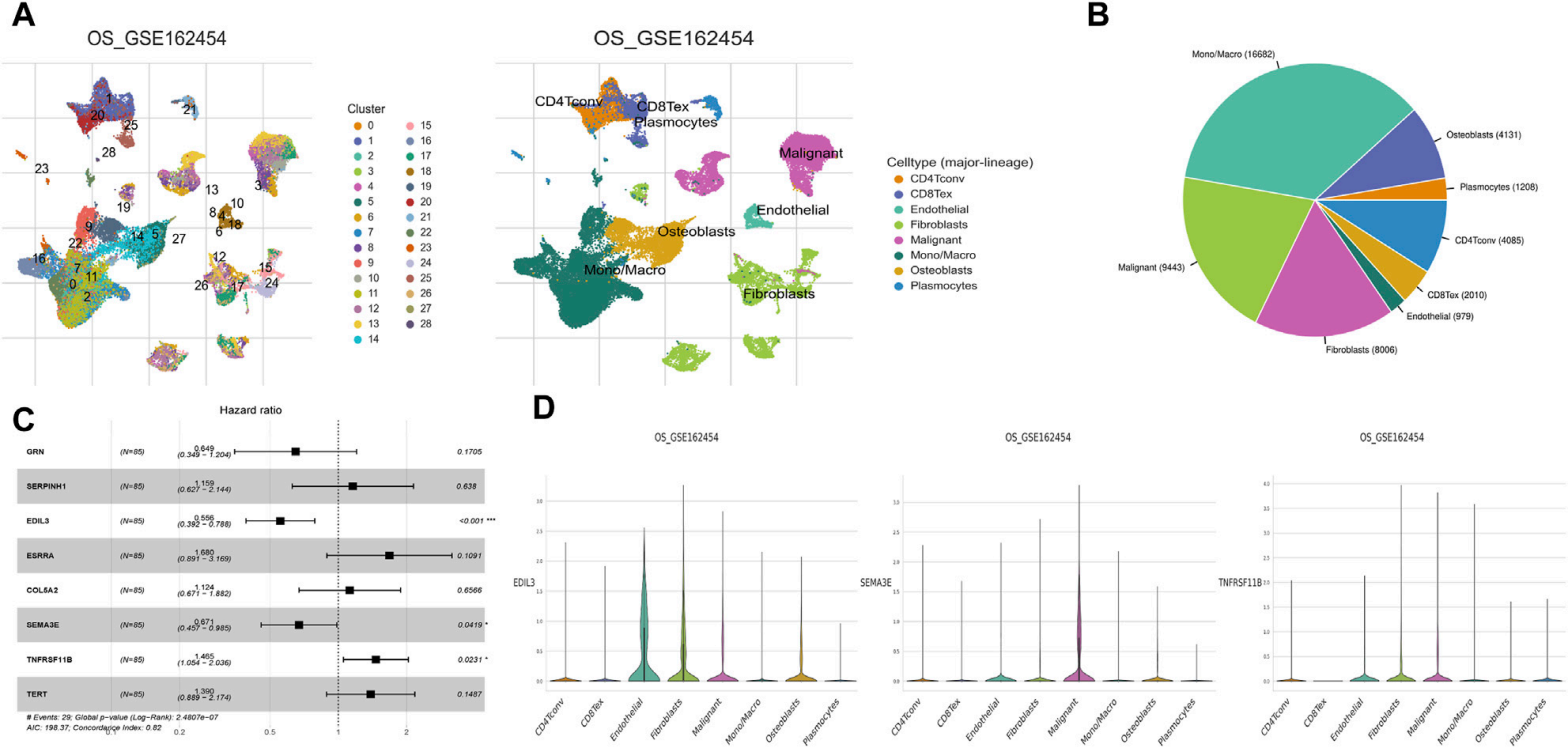
Single-cell landscape of hub genes. **(A)** UMAP plots displayed 28 main cell clusters and eight main cell types. **(B)** Pie chart displayed the immune cell composition of OS samples. Mono/Macro was found to dominate the tumour immune microenvironment. **(C)** Multivariate Cox regression identified three hub genes that affected the prognosis of OS, including EDIL3, SEMA3E, and TNFRSF11B. **(D)** Violin plots showing hub EDIL3, SEMA3E, and TNFRSF11B at the single-cell level in each of the eight main cell types.

The cellular heterogeneity in tumor tissues of OS patients was characterized using scRNA-seq data analysis. In the UMAP plot, a total of 28 main cell clusters were displayed. Followed all clusters annotated using markers, the UMAP representing all sequenced cells revealed eight main cell types: CD4Tconv, CD8Tex, endothelial, fibroblasts, malignant, Mono/Macro, osteoblasts, and plasmocytes (Figure 10A). Among these cell types, Mono/Macro was found to occupy the highest proportion in the tumor microenvironment (Figure 10B). Furthermore, three prognostic hub genes were screened, including EDIL3, SEMA3E, and TNFRSF11B (Figure 10C). Further analysis demonstrated different expression patterns of each gene in various cell types. EDIL3 showed a high expression level in endothelial cells and fibroblasts, while SEMA3E in malignant cells, and TNFRSF11B in fibroblasts and malignant cells (Figure 10D).

## Discussion

Osteosarcoma (OS) is a well-known malignant bone tumor with great harm in children and adolescents (Gilsenan et al., 2021; Rojas et al., 2021). Owing to this tumor’s high malignancy, OS patients continue to have an unsatisfactory survival rate, with over half dying from tumor cell metastasis and resistance to chemotherapy (Chou and Gorlick, 2006; Benjamin, 2020). Consequently, gaining a more profound understanding of the possible biomechanisms linked to the advancement of OS is especially crucial, thus creating novel therapies to improve the clinical outcomes for patients with OS. EMT, a cellular process, has been identified to be closely associated with the initiation and migration of cancers, including breast and bladder cancers (Chen et al., 2021; Kong et al., 2021). It has also been proved to result in drug resistance in lung and breast cancers (Luo et al., 2018; Tulchinsky et al., 2019). Moreover, many studies have demonstrated the connection between EMT and immunity in human cancers (Lou et al., 2016; Mak et al., 2016). Recently, there are studies showing that EMT plays a important role in the progression of OS, potentially elucidating why EMT leads to poorer clinical results in OS patients (Jiang et al., 2019). A multitude of distinct prognostic EMT-RGs have been investigated (Zhang Y. et al., 2019; Chen et al., 2019). Here, we explored the prognostic value of EMT-RGs by bioinformatics analysis and constructed created a novel EMT-Related risk signature. It has been shown that prognostic-related gene signatures from sequencing data play important roles in the identification of risk stratification and prediction of survival, developing precise treatment strategies for cancer patients (Gong et al., 2023).

Our research revealed that patients with OS can be divided into two distinct categories based on the EMT-Related DEGs, exhibiting significant differences in somatic mutations, immune responses, and possible mechanisms. The stromal score of the EMT-high subgroup was significantly higher, indicating that stromal cells inside the tumor microenvironment may be the source of EMT-RG expression in OS. This idea was also supported by earlier research on colorectal, urothelial, and OS cancers (Isella et al., 2015; Wang et al., 2018). The EMT-high subtype was related to positive clinical results along with an active immune reaction. Peng et al. (2020) revealed that a high EMT score was linked to significantly poor overall survival in OS patients, which was contrary to the result of this study. But judging by the results of the immune response, we could discover that immune cells, such as plasma cells and activated memory CD4 T cells, were increased in the EMT-high subtype, while resting dendritic cells were lower than the EMT-low subgroup. The anti-tumor immunity and immunosurveillance against cancer are beneficially mediated by plasma cells and CD4 T cells, and the enhancement of their responses may make cancer immunotherapies more effective (Wouters and Nelson, 2018; Yamamoto et al., 2020). By processing immune signals and presenting antigens to T cells, activated dendritic cells can initiate immunological cascades, which may explain lower levels of immunoreaction in patients with higher amounts of resting dendritic cells (Gardner and Ruffell, 2016; Hato et al., 2024). PDCD1LG2 was also discovered to be higher in the EMT-high subgroup by ICP expression analysis. It’s been established that improved general survival in hepatocellular carcinoma is linked to this gene expression (Lei et al., 2021). All these above could explain the better overall survival in the EMT-high subgroup to some extent. Therefore, we speculated that the prognosis of OS patients may be not directly related to the expression of EMT-RGs, but associated with the immune response accompanying the process of EMT, and active immune response may contribute to better clinical outcomes in OS patients.

To acquire a deeper insight into the possible pathways of EMT-RGs in OS development, functional enrichment studies were subsequently carried out. The results of GO analysis showed possible mechanisms in the progression of OS affected by EMT-RGs. To be specific, abnormal activation of B cells promoted by antigen binding, induce immunoglobulin production which bind to the corresponding receptors, leading to abnormal immune responses ultimately. In KEGG analysis, pathways mainly enriched were metabolic process related pathways, implying a coordinated interaction of these processes in OS. In light of the possible link between GO analysis outcomes and immune-related pathways, GSEA was employed in the two EMT-Related subgroups. And the results indicated a very close relationship between EMT-RGs and immunity in the occurrence and development of OS. These results could provide implications when developing new treatment methods for OS, especially immunotherapy.

Furthermore, we constructed a predictive risk signature using eight EMT-RGs, including GRN, SERPINH1, EDIL3, ESRRA, COL5A2, SEMA3E, TNFRSF11B, and TERT. GRN, by encoding granulin precursor, mainly controls the survival and differentiation of neurons, and is linked to immune, inflammatory, and stress reactions in the nervous system (Chu et al., 2023; Cai et al., 2024). SERPINH1, also known as HSP47, is noteworthy in the development of several kinds of human malignancies, including breast cancer, cervical cancer and other malignancies (Nagata et al., 1986; Yamamoto et al., 2013; Yoneda et al., 2020). Xia et al. (2024) revealed than SERPINH1 could enhance the malignancy of OS via PI3K-Akt signaling pathway. EDIL3 acts as a pro-angiogenic factor and associates with worse clinical outcomes of several cancers, such as gastric, breast and pancreatic cancers (Jiang et al., 2016; Kun et al., 2019; Zhang et al., 2020). There are studies suggesting that EDIL3 may promote EMT in cancer cells by facilitating autocrine or paracrine signaling (Gasca et al., 2020). ESRRA, full name estrogen related receptor alpha, is considered as an orphan nuclear receptor (Li FN. et al., 2021). Earlier research indicates a link between the over expression of ESRRA and unfavorable cancer outcomes, as it hastens the cancer cell proliferation and improves their ability to migrate and invade (Zhang L. et al., 2019; Wang L. et al., 2020). COL5A2 is crucial for regulating the immune system, promoting angiogenesis, and facilitating tumor metastasis (Ding et al., 2021). It was found by Han et al. (2022) that COL5A2 could prevent the malignant progression of OS. SEMA3E was found to play an important role in OS metastasis induced by UHRF1 overexpression. TNFRSF11B, also called osteoprotegerin (OPG), has been confirmed to participate in OS growth. Marley et al. (2015) revealed that OPG could increase proliferation in human derived OS cell lines. TERT, fully known as telomerase reverse transcriptase, is a catalytic subunit of telomerase, abnormal expression of which can activate the telomerase and play a key role in the cancer formation (Zou et al., 2020). A vitro study by Xie et al. (2023) indicated that inhibiting TERT may reduce the motility, metastasis, and proliferation of OS cells.

According to survival analyses, eight previously listed genes showed a strong correlation with the prognosis of OS patients and the high-risk individuals had a worse prognosis. The predictive precision of the risk model underwent additional validation by ROC curves. Moreover, multivariate Cox regression analysis provided convincing proof of the independence of the risk model. In order to improve the prediction of OS patients’ prognosis, we created a nomogram that incorporated clinical features such as gender, age, tumor-site, and metastatic situation. In the training cohort, the nomogram’s predictive performance was demonstrated with effectiveness, evidenced by survival rates at 1-, 3-, and 5-years, which further proved the risk model’s prediction effectiveness.

MSI score of tumor tissues can show how well ICB is working as a treatment, and the higher the score, the better the effects (Lichtenstern et al., 2020; Lin et al., 2020; Jiang et al., 2021). The high-risk group exhibited a higher MSI score in our research, suggesting that patients in this category may benefit more from ICB. However, upon comparing the immunological characteristics of the two risk groups, we discovered that patients with higher risk scores presented poorer immune infiltration. Additionally, we discovered the risk score was positively related with the expression levels of resting dendritic cells, while negatively correlated with activated memory CD4 T cells. Integrating all of these factors, we speculated that while high MSI scores would induce immune reactions, missing activated dendritic cells would eventually prohibit T cells from activation to efficiently attack cancer cells. A study by Pan et al. (2022) also has the similar speculation. The following TIDE analysis showed that the percentage of patients who responded to immunotherapy and those who did not shown any discernible variation. To summarize the above, we could suspect that OS patients may be not very sensitive to immunotherapy. In reality, OS is regarded as a “cold” tumor that may not respond well to ICP inhibitor therapy or be receptive to ICB (Wu et al., 2020; Li X. et al., 2021). Finally, upon conducting oncoPredict, it was discovered that patients in the high-risk subtype had lower IC50 values and greater sensitivity for five drugs, including vorinostat, lapatinib, VSP34_8731, I-BRD9, and NVP-ADW742. These findings may help guide individualized treatments for OS patients.

Data from scRNA-seq represents a novel method in cancer studies, aimed at identifying tumor cell composition and analyzing gene expression changes at the individual cell scale (Guo et al., 2024). This study revealed Mono/Macro as the predominant cell type in the tumor microenvironment, indicating their potential critical roles in the pathogenesis of OS. Further analysis of scRNA-seq data indicated that the cell types in which three hub genes highly expressed were not exactly same. These discoveries gain comprehensive insight on the molecular and cellular variations of OS, and have significant ramifications for developing novel treatment approaches that target particular cell types and genes. Nonetheless, more studies are required to corroborate these findings and ascertain their clinical relevance.

However, this study has several limitations. First of all, the training cohort of this study contains only 85 OS samples from the TARGET database, making the sample size small. Also, our model needs to be further validated using datasets outside of the GSE21257. Moreover, eight genes we have identified that may influence the prognosis of OS patients require further experiments *in vivo* and *in vitro* to elucidate their exact mechanisms of action. Nevertheless, our findings emphasize the significance of EMT-Related gene classifications in assessing the tumor immune microenvironment as well as predicting the prognosis of patients with OS. These findings not only contribute to the development of new treatment methods, but also help clinicians better predict the clinical outcomes of patients.

## Conclusion

In summary, our study analyzed the tumor immune microenvironment, immune response and biological functions in EMT-Related subtypes. And the prognosis of OS patients could be independently predicted by the risk signature constructed based on eight EMT-Related DEGs. Our results may give physicians novel perspectives into how to evaluate the prognosis of OS patients and develop more customized and efficient therapy regimens for OS patients, yet further study is still needed to validate our findings.

## Data Availability

The original contributions presented in the study are included in the article/Supplementary Material, further inquiries can be directed to the corresponding author.
